# Biochemistry and Future Perspectives of Antibiotic Resistance: An Eye on Active Natural Products

**DOI:** 10.3390/antibiotics13111071

**Published:** 2024-11-11

**Authors:** Giulia Caioni, Carolina Pérez Reyes, Davide Laurenti, Carmen Chiaradia, Enrico Dainese, Roberto Mattioli, Daniel Di Risola, Eleonora Santavicca, Antonio Francioso

**Affiliations:** 1 Department of Bioscience and Technology for Food Agriculture and Environment, University of Teramo, 64100 Teramo, Italy; gcaioni@unite.it (G.C.); edainese@unite.it (E.D.); 2 Department of Biochemistry, Microbiology, Cell Biology and Genetics, Instituto Universitario de Bio-Orgánica “Antonio González”, University of La Laguna, 38206 San Cristobal de La Laguna, Spain; cpreyes@ull.edu.es; 3 Department of Biochemical Sciences “A. Rossi Fanelli”, Sapienza University of Rome, 00185 Rome, Italy; davide.laurenti@uniroma1.it (D.L.); carmen.chiaradia97@gmail.com (C.C.); roberto.mattioli@uniroma1.it (R.M.); daniel.dirisola@uniroma1.it (D.D.R.); 4 Farmacia D’Onofrio, San Pio delle Camere, 67020 L’Aquila, Italy

**Keywords:** natural bioactive compounds, antibiotics, biological chemistry, pharmacology, phytochemistry, antibiotic resistance, plants, one health

## Abstract

Antibiotic resistance poses a serious threat to the current healthcare system, negatively impacting the effectiveness of many antimicrobial treatments. The situation is exacerbated by the widespread overuse and abuse of available antibiotics, accelerating the evolution of resistance. Thus, there is an urgent need for novel approaches to therapy to overcome established resistance mechanisms. Plants produce molecules capable of inhibiting bacterial growth in various ways, offering promising paths for the development of alternative antibiotic medicine. This review emphasizes the necessity of research efforts on plant-derived chemicals in the hopes of finding and creating novel drugs that can successfully target resistant bacterial populations. Investigating these natural chemicals allows us to improve our knowledge of novel antimicrobial pathways and also expands our antibacterial repertoire with novel molecules. Simultaneously, it is still necessary to utilize present antibiotics sparingly; prudent prescribing practices must be encouraged to extend the effectiveness of current medications. The combination of innovative drug research and responsible drug usage offers an integrated strategy for managing the antibiotic resistance challenge.

## 1. Introduction

Antibiotics were the most important and influential medical achievement of the 20th century, playing an indispensable role in ensuring both human and animal health. These substances, also referred to as antimicrobials, are capable of inhibiting bacterial proliferation (bacteriostatics) or killing bacteria (bactericides) to cure infections. Antibiotics vary significantly in their bactericidal mechanisms and chemical compositions, reflecting the absence of a universally effective antibiotic capable of targeting all bacterial types [1].

Before the advent of antibiotics, the knowledge of microbes and infectious diseases was inadequate. The methods of curing and preventing the transmission of contagious diseases were ineffective, resulting in the consequent deaths of millions of people. For instance, in the 14th century, the bubonic plague pandemic also known as the “Black Death” caused millions of deaths in Europe, due to the lack of available antibiotics against *Yersinia pestis* at the time [2].

In 1676, through the discovery of microscopic organisms, Antonie van Leeuwenhoek sowed the seeds for the development of antibiotics. It was not until the late 19th century that significant independent research on bacteria was conducted by Louis Pasteur, who studied *Bacillus anthracis*, and Robert Koch, who focused on *Mycobacterium tuberculosis* [3]. The work of these bacteriologists established a link between specific bacterial species and infectious diseases, setting the stage for the modern era of microbiology.

The first antibiotic, mycophenolic acid, was discovered in 1893 by the Italian microbiologist Bartolomeo Gosio, from the mold *Penicillium glaucum*. In 1909, Paul Ehrlich discovered arsphenamine (Salvarsan), i.e., the first synthetic antibiotic derived from arsenic effective against *Treponema pallidum*, i.e., the agent of syphilis. During World War I, Prontosil, an antibiotic with a broad spectrum of action, was used to treat wounded soldiers. In 1928, the bacteriologist Alexander Fleming discovered that the fungus *Penicillium notatum* inhibited the growth of colonies of *Staphylococcus aureus*, hypothesizing that the fungus could produce a compound that inhibited the bacteria. In 1929, he isolated the molecule that he called penicillin, marking the discovery of the first antibiotic as we know them nowadays [4].

Antibiotics experienced a glorious era between the 1940s and 1970s when more than 20 chemical classes were derived from hundreds of species of bacteria and fungi. The last time a new class of antibiotics was discovered and patented was in 1987, and refers to oxazolidinones, which introduced a novel mechanism based on blocking bacterial protein synthesis [5]. Recently, the development of new antibiotics has continued slowly, and only 5 of the 20 pharmaceutical companies that contributed to antibiotic research in the 1970s still remain active [6].

In the development of therapeutic and preventive strategies within medical and veterinary domains, it is crucial to acknowledge the concurrent existence of two interrelated yet distinct phenomena: antimicrobial resistance (AMR) and antibiotic resistance (ABR) [7,8,9]. AMR occurs when a microorganism becomes resistant to an antimicrobial drug previously effective against it. ABR only refers to bacteria developing resistance against antibiotics. Thus, a bacterium is considered antibiotic-resistant when the antibiotic meant to eradicate it or to stop its growth is no longer effective. The term “ESKAPE” encompasses the six pathogenic bacteria known for their virulence and high antibiotic resistance: *Enterococcus faecium*, *Staphylococcus aureus*, *Klebsiella pneumoniae*, *Acinetobacter baumannii*, *Pseudomonas aeruginosa*, and *Enterobacter* spp. These ESKAPE pathogens are associated with high mortality risk, tending to become resistant to one or more antibiotics if used in combination, due to the selection of resistant strains and also to horizontal resistant gene transfer [10].

The proliferation of AMR and ABR is primarily attributed to the excessive and inappropriate use of antibiotics across both medical and agricultural domains. In the agricultural sector, particularly within animal husbandry, antibiotics are routinely administered not only for therapeutic purposes but also as growth promoters and prophylactics, aimed at enhancing productivity and consumer goods output. Furthermore, these compounds often persist in the environment, thereby exerting selective pressure on microbial communities and facilitating the emergence and dissemination of resistant strains. This environmental persistence represents a significant factor in the global spread of AMR [11].

After outlining the background and then the key issues related to antibiotic resistance, it is evident that further research on this topic is required, with an emphasis on substitute remedies. After reviewing the main pharmacological targets and the basic mechanisms underlying antibiotic and antimicrobial resistance, we will discuss how natural compounds may be a viable substitute for the use of conventional antibiotics in this review, thus highlighting the presence of relevant molecules in the most common matrices.

## 2. From Cure to Concern: Tracing the Pathway to Antibiotic Resistance

### 2.1. Mechanisms of Antibiotic Actions

**Figure 1:**
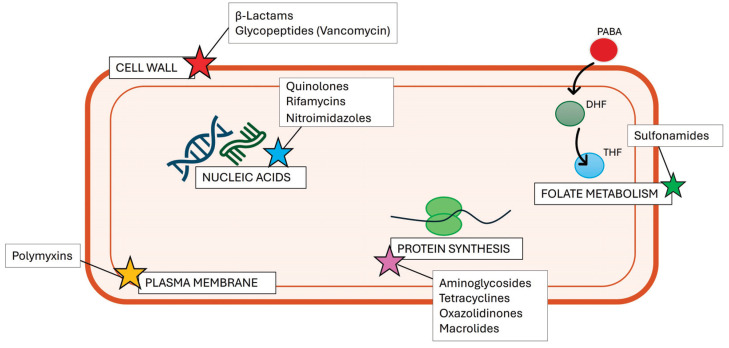
Schematic illustration of bacterial targets of currently used antibiotics. Abbreviations: PABA, para-aminobenzoic acid; DHF, dihydrofolate; THF, tetrahydrofolate.

The term “antibiotic” refers to a natural or synthetic drug capable of blocking or at least slowing the proliferation of bacteria. Antibiotics exert their effects by targeting different bacterial structures and can be classified according to their mechanisms of action. The most important groups of antibiotics and their main targets are presented and summarized in Figure 1 and Table 1.

### 2.2. The Development of Antibiotic Resistance

Shortly after penicillin was introduced in the early 1940s, the first cases of antibiotic resistance were documented, since scientists noticed that certain bacterial strains were no longer susceptible to the antibiotic challenge. However, even before antibiotics were widely used, Nobel laureate Alexander Fleming had foreseen the possibility of antibiotic resistance, and during his 1945 Nobel Prize acceptance speech, he mentioned that antibiotic misuse could result in resistance [25].

Microorganisms exhibit the fundamental ability to adapt over time, with their main goals being replication, survival, and spreading as quickly as possible. They adapt to the environment and evolve in ways that ensure their survival. The occurrence of random mutations during replications may allow some bacterial populations to survive in the case of exposure to an antibiotic, at the expense of the other antibiotic-sensitive populations. This is the natural process through which bacterial resistance develops [26].

There are two primary forms of antibiotic resistance: intrinsic and acquired. Intrinsic resistance is innate in organisms [27]. For instance, mycoplasmas lack a cell wall, so they are naturally resistant to antibiotics that act to lyse the bacterial cell wall [28]. On the other hand, acquired resistance derives from the selection of bacterial strains whose genetic changes enabled them to survive in the presence of a specific antibiotic [29]. Moreover, drug-resistant bacteria can transmit a copy of their genes to other non-resistant strains; resistance genes can be carried on plasmids or other types of mobile elements, which can spread to bacteria of different genera and species. This is called horizontal resistance and is one of the main causes of antibiotic resistance [30]. This kind of resistance is achieved through the mutation of the drug targets, the production of enzymes that inactivate the antibiotics, reduced permeability, and/or the active efflux of drugs via pumps. Table 2 summarizes the main mechanisms of resistance [31,32,33].

Antibiotic resistance in bacterial strains is classified according to its extent as monoresistance, multidrug resistance (MDR), or total drug resistance (also defined as extensive drug resistance, XDR). Initially, this classification referred exclusively to acquired, rather than intrinsic, resistance. However, establishing consistent terminology remains challenging, as multiple factors must be considered. These definitions were initially applied primarily to characterize resistance patterns in Mycobacterium tuberculosis strains to ensure effective treatment for tuberculosis (TB) patients. In this context, monoresistant strains were defined as those resistant to only one first-line anti-TB drug, while MDR strains demonstrated resistance to at least both isoniazid and rifampicin. XDR strains exhibited MDR with additional resistance to any fluoroquinolone and at least one of the three second-line injectable drugs (capreomycin, kanamycin, or amikacin) [37]. Today, this classification framework has broader applications, including *S. aureus*. Methicillin-resistant *S. aureus* (MRSA) serves as an important paradigm of common multidrug-resistant organisms (MDROs), demonstrating resistance not only to methicillin but also typically to aminoglycosides, macrolides, tetracyclines, chloramphenicol, and lincosamides [38]. For accurate categorization, an isolate should be tested against a comprehensive panel of antimicrobials tailored to the specific bacterial species [39].

The natural and acquired mechanisms used by bacteria to survive are not the only factors responsible for antibiotic resistance. Several causes have led to an increase in drug-resistant strains: (1) the inaccurate or inappropriate diagnosis of an infection; (2) the onset of healthcare-associated infections (HAIs) [40]; (3) antibiotic environmental contamination; (4) the human-to-human transmission of resistant bacteria; and (5) the overuse of antibiotics in agricultural and farming practices [41].

During the diagnostic process, healthcare providers may prematurely resort to broad-spectrum antibiotics as a precautionary measure rather than opting for narrow-spectrum antibiotics better suited to the specific type of infection. Moreover, without adequate laboratory testing, it is challenging to determine whether an infection is bacterial or viral. Prescribing antibiotics for a viral infection is futile, as they do not affect viruses and may foster resistance. For instance, seasonal fevers are typically viral; using antibiotics to treat them could exacerbate the patient’s condition.

## 3. Natural Antibacterial Products: A Viable Alternative

Natural products are a promising source for drug discovery and development because of their remarkable chemical diversity and range of biological effects. The plant kingdom deserves special attention as it offers numerous compounds with antibacterial properties that have demonstrated efficacy in treating bacterial infections [42,43].

Several factors support the viability of natural compounds as alternatives to synthetic substances. Most of them can act through multiple pathways simultaneously and exhibit synergistic effects when used in combination with conventional antibiotics [44] or in mixtures, resulting in enhanced antibacterial activity compared to individual components [42,45]. An indication of the importance of these compounds can be inferred from the observations made by Newman and Cragg (2007). Specifically, in exploring the role of natural products as a source of new drugs over 25 years (1981–2006), it was observed that, in the antibacterial field, of the 109 new chemical entities (NCEs) approved by the FDA, 64 were derived from natural products [46].

**Figure 2:**
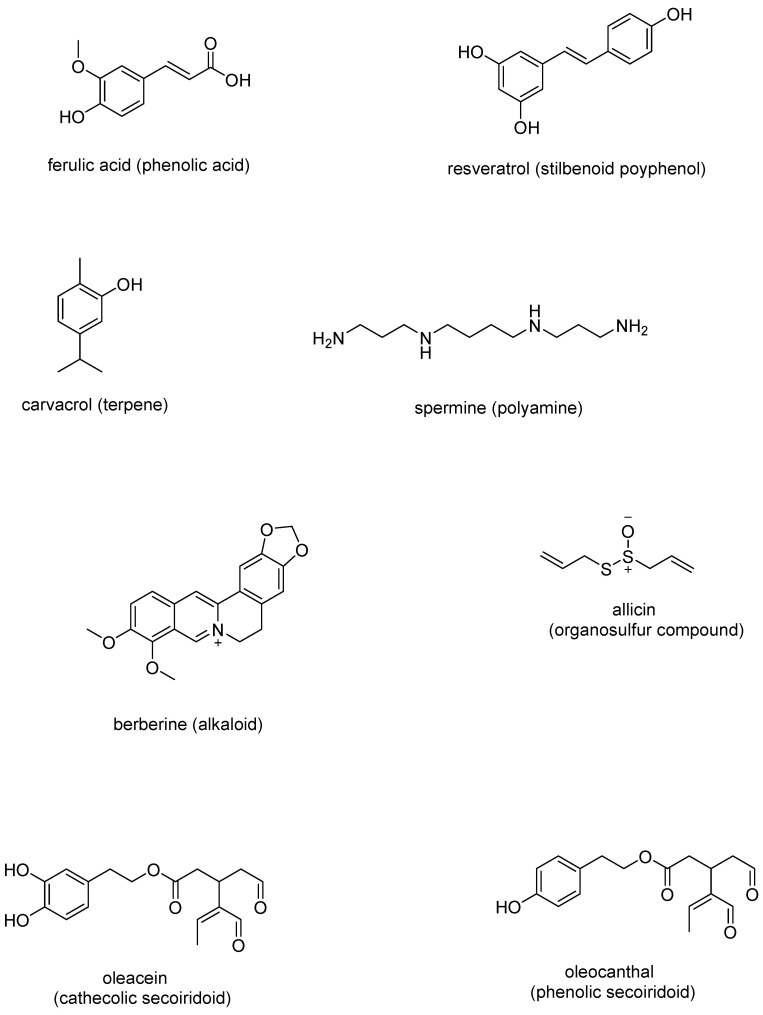
Chemical structures of representative natural antibiotic compounds.

The antibacterial efficacy of plants can be attributed to various constituents and their specific concentrations. Notable among these are classes of phytochemicals such as phenols, polyphenols, terpenoids, essential oils, alkaloids, sulfur-containing compounds, and their combinations (Figure 2) [42,47].

Phenolic compounds serve as secondary metabolites in plants, playing roles in defense, adaptation mechanisms, and pigmentation. These compounds offer health benefits and have shown efficacy against various diseases like cardiovascular disease, cancer, and diabetes [48]. Due to the structural diversity within this class, their antibacterial mechanisms are multifaceted, involving membrane permeability or instability and the inhibition of extracellular enzymes [49,50,51,52,53]. Plant phenolic compounds provide a promising alternative to combat antibiotic resistance due to their distinct mechanisms of action compared to conventional antibiotics [54]. Phenolic compounds’ antibacterial effects have been demonstrated in vitro. For instance, in a study targeting periodontitis-causing bacteria, two phenolic compounds, pyrogallol and pyrocatechol, displayed efficacy with minimum inhibitory concentration (MIC) values ranging from 2 to 2500 μg/mL for pyrogallol and 4 to 312 μg/mL for pyrocatechol [55]. Further studies on both Gram-positive and Gram-negative bacteria have identified pyrogallol as the most potent of the phenolic compounds tested, highlighting the significance of hydroxyl groups in their antibacterial activity [56]. The interaction of phenolic compounds with bacterial proteins or cell walls and modifications to the synthesis processes of macromolecules (nucleic acids) or energy production are some of the diverse mechanisms of action. Furthermore, some properties of polyphenols appear to increase their effectiveness. Indeed, the planar structure and hydrophobicity of these molecules allows them to interact with the DNA, interfering with the processes of replication and repair. Also, the presence of hydroxyl groups results in the creation of hydrogen bonds with the nitrogenous bases [57].

Among phenolic and polyphenolic compounds, there is a large class of molecules named flavonoids. Isoflavones, flavonols, anthocyanins, and flavanones are a part of this large class of compounds. In particular, anthocyanins demonstrate antibacterial properties, and thus, the plausible mechanisms by which these effects are exerted have been evaluated. Studies on the antibacterial properties of anthocyanins using *S. aureus* bacteria as a model revealed that anthocyanin treatment not only prevented the growth of the bacterial colonies but also slowed down the growth and activity of the bacterial biofilm, with stronger effects at higher concentrations. In addition, anthocyanins have been able to cause a massive efflux of K^+^, which inhibits the Na-K ATPase and consequently causes the death of the bacterial cells. Finally, anthocyanins can inhibit protein synthesis, halting the process of bacterial colony growth [58]. Catechins are compounds found in some types of tea (such as green tea) and belong to the flavan-3-ol family. Over the past few decades, they have been shown to inhibit the in vitro growth of certain bacterial strains. Specifically, epigallocatechin gallate was able to inhibit by 90% the bacterial growth of Helicobacter pylori colonies (including strains resistant to metronidazole and clarithromycin) when administered at a concentration of 100 µg/mL. However, this effect was not observed in Escherichia coli colonies when administered at 25 µg/mL. In this case, some virulence factors were reduced, such as biofilm formation or bacterial motility [59].

Within the stilbene family of polyphenols, resveratrol is well known for its immune system- and neurodegenerative system-protective capacities as well as its antioxidant activity [60]. Plants such as grapevines, pines, bananas, beans, and even pomegranates, peanuts, soybeans, and others contain significant amounts of resveratrol. Additionally, significant quantities of this molecule are found in red wine, which typically contains about 1.9 mg/L of resveratrol in its trans configuration [61]. Numerous investigations have exhibited its ability to reduce the formation of biofilms in a range of bacterial species, including *Vibrio cholerae* [62], *E. coli* [63], and *S. aureus* [64]. Additionally, it has been shown to interfere with quorum sensing [65], a crucial mechanism for bacterial cell communication, as well as the synthesis of microbial toxins [66], which are known to have a substantial impact on the virulence of the bacterium. Moreover, resveratrol can inhibit ATP synthase in *E. coli* bacteria and fragment their DNA. Most notably, resveratrol inhibits the *ftsZ* gene, which is responsible for bacterial cell division, thus preventing colony growth [67]. In addition to its pleiotropic beneficial effects in the case of neurodegenerative diseases [68], the polyphenol curcumin (extracted from *Curcuma longa*) showed antibacterial activity in in vitro studies conducted on *S. aureus*, *E. coli*, *Enterococcus faecalis*, and *P. aeruginosa* [69]. The underlined mechanisms seem to be linked to the ability to inhibit bacterial adhesion and growth [69]. Detailed studies conducted by Rai and colleagues (2008) have shown that curcumin was capable of affecting the assembly and stability of FtsZ protofilaments, which play a crucial role in cytokinesis. Disruption in these protofilaments is lethal for bacteria [70].

Another subgroup, phenolic acids, including caffeic acid, gallic acid, and ferulic acid, exhibit notable antibacterial activity. Comparative studies have shown caffeic acid’s superior activity against *S. aureus* and *E. coli* compared to ampicillin [71]. Gallic acid, a polyphenol, displays significant antibacterial activity, particularly against Campylobacter species, attributed to its disruptive effect on the cell wall structure [72]. Some natural substances, such as ferulic acid, can amplify the effects of certain antibiotics. This compound, found in rice, wheat, oats, artichokes, peanuts, and nuts [73], has been shown to exhibit antibacterial activity against both Gram-positive and Gram-negative bacteria. Ferulic acid may boost the antibacterial effects of quinolones by producing ROS. In fact, there is evidence of a combined effect when ferulic acid is used alongside ciprofloxacin or gemifloxacin. It has been demonstrated that when used as a co-treatment with ciprofloxacin and gemifloxacin against *A. baumannii*, ferulic acid induces a higher production of superoxide anion, which decreases glutathione levels, thus inducing cell death [74].

Terpenoids, comprising a diverse group of hydrocarbons, including monoterpenes, sesquiterpenes, diterpenes, and triterpenes, have demonstrated antibacterial activity [75] through mechanisms such as affecting oxygen absorption and oxidative phosphorylation, thereby disrupting cellular respiration [76]. Monoterpenes like carvacrol, thymol, menthol, and geraniol exhibit antibacterial effects against both Gram-positive and Gram-negative bacteria [77]. Geraniol, unique among monoterpenes, shows efficacy against Gram-negative MDR bacteria by inhibiting efflux pumps [78]. Sesquiterpenes, exemplified by farnesol, display potent antibacterial activity, with studies indicating synergistic effects when combined with antibiotics against various bacterial strains [79,80]. Moreover, a recent study by Ivanova et al. (2022) also demonstrated the positive effects of a nanoformulation of farnesol in preventing the development of drug resistance in *S. aureus* [81]. Diterpenes, such as salvipisone and aethiopinone extracted from the hairy roots of Salvia sclarea, exhibit bactericidal or bacteriostatic effects against methicillin-resistant *S. aureus* (MRSA) and Staphylococcus epidermidis (MRSE) [82]. Triterpenes, including oleanolic acid, betulinic acid, and amyrin, demonstrate antibacterial activity against resistant strains of *S. aureus*, often enhancing the activity of conventional antibiotics [83].

Essential oils, volatile substances produced by plants, possess antibacterial properties attributed to constituents like terpenes and phenylpropanoids [84,85,86]. A study by Sakkas and colleagues (2018) tested the efficacy of essential oils (made from basil, chamomile, tea tree, and thyme) against Gram-positive bacteria, revealing that commercially available formulations from origanum had the best antimicrobial activity, followed by thyme, tea tree, basil oil, and chamomile [87].

Alkaloids, a diverse group with over 18,000 representatives, exert antibacterial effects through various mechanisms, including the inhibition of cell division, respiration, bacterial enzymes, membrane damage, and the modulation of virulent genes [88]. Examples include pergularinins, thylophorinidines, ugeremine, and alkyl methyl quinolone (highly strong and selective against *H. pylori* [89]). Natural alkaloids can act in different modes of action [45], demonstrating them to be a valuable resource against antibiotic resistance. Berberine, an isoquinoline quaternary alkaloid, is a secondary metabolite in Berberis species plants and was demonstrated to inhibit the growth of MRSA isolated from patients with bloodstream infections, damaging the cell wall and membranes [90]. The action of sanguinarine (extracted from *Macleaya cordata*) was revealed to be specific against *S. aureus*, in which it induces oxidative damage and interferes with the permeability and integrity of the membrane [91]. Also, piperine, found in black pepper, has been tested for its antimicrobial activity, demonstrating a minimum inhibitory concentration (MIC) below 100 μg/mL against *S. aureus* and *Salmonella* spp. [92]. Capsaicin possesses a multitude of health-related properties, including anti-inflammatory, analgesic, and cardioprotective effects. Additionally, it can exhibit both bacteriostatic and bactericidal effects against both Gram-positive and Gram-negative bacteria. Further effects of capsaicin involve the reduction in toxin release and the inhibition of biofilm formation [93]. This latter property is particularly significant as bacterial biofilms, in fact, made up of proteins, carbohydrates, extracellular DNA, and lipids, create a protective and viscous matrix around bacteria and act as a barrier [94], enhancing the already acquired resistance of bacteria [95].

Recently, other molecules have emerged that show pronounced antibacterial qualities, such as the secoiridoids found in olive oil. Oleocanthal and oleacein, for example, are two polyphenols belonging to the secoiridoid class, produced during the malaxation and production phases of extra virgin olive oil (EVOO), which seem to play an important role in counteracting bacterial growth or biofilm production. Specifically, oleocanthal and oleacein, as well as polyphenolic extracts from EVOO enriched with these two polyphenols, appear to be capable of inhibiting the growth of *Chlamydia trachomatis* at very low concentrations [96].

Furthermore, the antimicrobial activity of these polyphenols also seems to be effective against numerous bacterial strains resistant to various antibiotics, both Gram-positive and Gram-negative. In particular, polyphenolic extracts from EVOO enriched with oleocanthal and oleacein show the highest antibacterial activity against various clinical strains of *S. aureus*, while purified oleacein is more effective against several clinical strains of *E. coli*, *P. aeruginosa*, and *K. pneumoniae* [97].

Finally, it has been recently demonstrated that a mixture of oleocanthal and oleacein at a concentration of 2.5 mM each is able to inhibit the growth and adhesion of *P. aeruginosa* biofilms. The same effect is obtained when these substances are administered individually but at a higher concentration (5 mM) [98]. On the contrary, other polyphenols present in EVOO, such as tyrosol and hydroxytyrosol, derived from the degradation of oleocanthal and oleacein, respectively, show only a mild antimicrobial effect, suggesting the importance of the dialdehyde function to exert these effects [99].

In addition to the prominent classes mentioned earlier, the antibacterial efficacy of the following compounds has been thoroughly studied and documented: polyamines, isothiocyanates, glucosides, and thiosulfinates. Polyamines, as observed by Kwon and Lu, possess the intriguing property of augmenting the sensitivity of *P. aeruginosa* to various antibiotics such as chloramphenicol, nalidixic acid, and erythromycin, among others [100]. Among the isothiocyanates, sulforaphane, a well-known representative, has demonstrated antibacterial effects against *H. pylori* both in vitro and in vivo [101,102]. In terms of glucosides’ antibacterial potential, research conducted by Soulef investigated the effectiveness of an extract from the root of Glycyrrhiza glabra against three bacterial strains (*E. coli*, *P. aeruginosa*, and *S. aureus*), obtaining results only against *E. coli* [103]. Thiosulfinates, on the other hand, exhibit both bacteriostatic and bactericidal effects. The diallylthiosulfinate compound allicin is primarily responsible for the broad-spectrum antimicrobial properties of garlic (*Allium sativum*). It acts by binding to thiol groups and deactivating essential bacterial enzymes. Allicin exerts its activity at micromolar concentrations against Gram-positive and Gram-negative bacteria, including antibiotic-resistant strains [104,105,106]. The effectiveness of garlic was also tested in comparison to the antibiotic metronidazole in the treatment of bacterial vaginosis, proving to be a valid alternative especially for those patients who cannot tolerate conventional treatments [107]. 

The main compound classes discussed in the text, along with their sources and primary activities, are summarized in Table 3.

According to the findings, results from in vitro and in vivo analyses and studies demonstrate the great potential of these compounds. However, beyond efficacy, a crucial step is assessing such preparations’ safety. Recently, a study investigated the safety of a cocktail of natural products which in vitro showed the ability to counteract biofilm formation by resistant bacteria with a promising safety profile. Specifically, this was a Phase I clinical trial, including 109 volunteers, which assessed the safety of the preparation when applied to healthy skin, supporting further investigation into its clinical potential for treating wound infections [108]. Other recent clinical trials with promising results include evaluations of the antibacterial potential of a gel formulation derived from *Nigella sativa* L. seeds against acne-causing bacteria and the antibacterial efficacy of α-viniferin against *S. aureus* [109,110]. The first study demonstrated encouraging results regarding the stability of the tested formulations and their ability to inhibit the growth of *S. aureus* and *P. acnes* [109]. The second study confirmed the safety profile of α-viniferin as a topical agent and its effectiveness in reducing *S. aureus* levels in the nasal passage while preserving the nasal flora [110].

## 4. Approaches to Counteract Antibiotic Resistance

The World Health Organization (WHO) has considered antibiotic resistance one of the three most important threats to public health in the 21st century, along with climate change and the tendency to refuse vaccines. Antibiotic resistance is an emerging problem as there is a rapid loss of efficacy from an increasingly large number of drugs and the reduced development of new antibiotic molecules.

Environmental contamination plays a crucial role in this problem, since antibiotics can leach into water bodies and subsequently into the soil through human and pharmaceutical industry waste. This exposure induces resistance in environmental bacteria, which can then be transmitted to the bacteria that inhabit humans [111]. In the 1960s, the excessive use of antibiotics in livestock also led to resistance in bacteria residing within farm animals. These bacteria were transmitted to humans via zoonotic pathways through direct contact or food consumption. Finally, human-to-human transmission can occur within healthcare facilities, with direct or indirect contact [112,113]. The misuse of antibiotics, deriving from improper use or incorrect dosages, further contributes to the development of resistance. Non-adherence to prescribed treatment regimens can promote resistance. Efforts are being made towards antimicrobial stewardship to optimize antibiotic use and avoid unnecessary applications. In agriculture and livestock farming, there has historically been an excessive and unnecessary use of antibiotics [114]. Often, multiple antibiotics are administered simultaneously in the hope that one will be effective, thereby increasing the selective pressure on bacteria and promoting the emergence and proliferation of resistant strains. Additionally, inadequate hygiene in preventive practices and the lack of diagnostic testing that could guide precise prescribing exacerbate antibiotic resistance.

The WHO has established a Global Action Plan that is based on five fundamental strategic objectives: (1) to increase the knowledge of antibiotic resistance through education and communication; (2) to strengthen surveillance and research; (3) to reduce infections through effective health, hygiene, and prevention measures; (4) to optimize the use of antimicrobials in human and animal medicine; and (5) to increase investment in new drugs, diagnostic tools, and vaccines to prevent and treat infectious diseases with safer and longer-lasting drugs [115].

The SARS-CoV-2 pandemic has exacerbated the resistance of bacteria to antibiotics, as there has been inappropriate use of the drugs themselves. As many as 70% of COVID-19 patients were treated with at least one antibiotic for therapeutic purposes [116,117]. During the pandemic, the WHO discouraged the use of antibiotics for mild cases while recommending them for severe cases. This inappropriate use of drugs during COVID-19 has aggravated the threat of antimicrobial resistance. A systematic review by Langford and colleagues (2022) showed the role of the pandemic in accelerating the development and spread of antibiotic resistance, especially among Gram-negative bacteria in hospitals [118].

**Figure 3:**
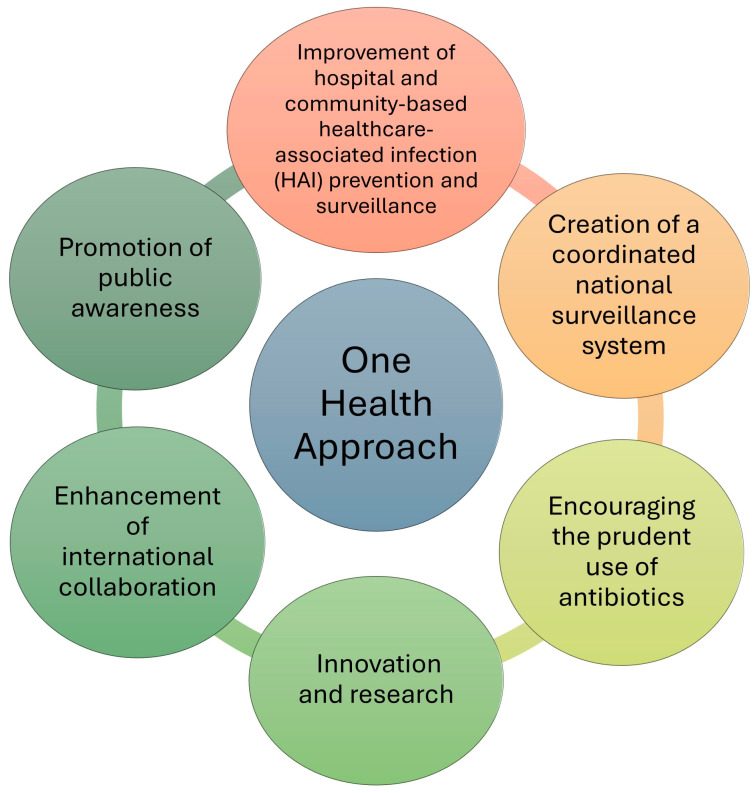
Main strategic guidelines characterizing one health approach for combating antibiotic resistance in Italy (PNCAR 2022–2025).

The challenge of antibiotic resistance is global and within each territory, there is the involvement of a wide range of sectors. This approach is known as “One Health”, based on the integration of different disciplines, aimed at the design and implementation of programs, policies, and research aimed at achieving better levels of global health. The one health healthcare model also believes that the health of humans, animals, and the environment are interconnected. The areas of work in which this approach is relevant include food safety and zoonosis control. In Italy, the National Plan for Combating Antibiotic Resistance (PNCAR) 2022–2025 was developed to provide strategic guidelines to address the problem in the coming years, referring to a multidisciplinary approach [119] (Figure 3).

### New Strategies

As concerns about antibiotic resistance continue to grow, new strategies for treating antibacterial infections are needed. Several new small antibiotic molecules are in preclinical testing and development phases. Antimicrobial stewardship, new antibiotic molecules, and delivery mechanisms must be integrated and combined into treatments that combat infection, prevent resistance, and maintain normal microbiota. One of the most promising methods is the system of administering a multidrug cocktail to counteract antibiotic resistance. The combination of antibiotics with adjuvants represents the main approach to addressing the problem. Among the groups recognized as antibiotic adjuvants are tranquilizers, antihistamines, anti-inflammatory drugs, etc. A combination of drugs may at the same time encourage the development of drug resistance despite an initial appearance of bypassing resistance mechanisms. In addition, there is growing promotion of the study of innovative approaches, such as the development of vaccines. Research on vaccines directed against antibiotic-resistant bacteria is being encouraged to prevent infection, transmission, and disease specifically, without having to resort to antibiotics [120].

In this regard, in addition to representing a viable alternative, natural compounds could represent a platform for the development of effective new drugs, as proposed by Rossiter et al. (2017). They indicated synthetic methodologies aimed at reengineering natural products into potent antibiotic agents. These strategies included combinatorial chemistry, which typically produces mainly sp2-hybridized molecules, approaches to create diverse libraries with complex molecular frameworks, and modifications to previously identified antibiotic compounds [121].

More and more attention is being paid to possible alternative targets. Many advantages of using bacteriophage therapy over chemotherapy have been claimed and it seems to be a potential treatment to replace antibiotics [122]. During this procedure, phages transfer genes that encode for antimicrobials or harmful antimicrobials into the target bacteria. The first step in the infection by the lytic phage is the adsorption to specific receptors on the surface of the bacterial host. These receptors can be found on Gram-positive or Gram-negative cell walls, on polysaccharide capsules, and even on appendages such as pili and flagella. After adsorption, the virus will introduce its genetic material into the host from the capside.

The CRISPR-Cas system is known as one of the recent methods to combat antibiotic-resistant strains [123]. The programmable nuclease Cas of this system, if used against bacterial genomic sequences, can be lethal or contribute to reducing antimicrobial resistance in bacteria. In this context, various approaches can be used, as is well explained in the review by Kadkhoda et al. [124]. Gene- or pathogen-focused approaches target either the resistance gene (typically located on an episomal plasmid) or specific regions of the bacterial genetic material, ultimately leading to the death of the pathogenic strain [124]. However, there are several limitations to the use of CRISPR-Cas approaches, such as bacteria developing resistance mechanisms mediated by anti-CRISPR (Acr) genes. Certain Acr genes were first discovered in virulent *P. aeruginosa* strains and can be transmitted to other *P. aeruginosa* strains through conjugation, effectively inhibiting CRISPR-Cas antibacterial action [125]. These same genes can be utilized to suppress bacterial immune responses. This novel approach is known as phage therapy: phages with Acrs can be engineered to target specific bacteria [124]. This allows for a modular strategy in which Acr activities are customized to various bacterial defenses. For example, the use of engineered bacteriophages (EATPs) for treating MDR bacteria was recently investigated in a study by Qin and colleagues (2022). The results demonstrated EATPs’ effectiveness in suppressing the growth of clinical isolates of MDR *P. aeruginosa* both in vitro, using human embryonic kidney 293 (HEK293T) cells, and in vivo, utilizing mouse respiratory infection models [126].

Microbiome-derived substances are an expanding class of non-traditional antibiotics under research and development, with promising applications in antimicrobial therapy [127]. Microorganisms that inhabit the same environmental niches in human microbiomes use distinct strategies to gain an advantage over competitors. The human microbiome produces many metabolites (lipids, oligosaccharides, amino acids, non-ribosomal peptides, and ribosomal peptides) with antioxidant, cytotoxic, and immunomodulatory effects. However, the human microbiome also contains numerous natural chemicals with antibacterial properties from various bacterial phyla. Among these, ribosomally produced peptides, also known as bacteriocins, were first described in 1925 [128]. Bacteriocins have negligible toxicity in human cells, and bacteria are unlikely to acquire resistance to them. Mulkern and colleagues (2022) have reported on the therapeutic potential of microbiome-derived peptides against bacterial infections [129]. Antimicrobial peptides from the rumen microbiome showed therapeutic promise against seven clinical strains of *P. aeruginosa*, with low damage to human lung cells [129].

RNA therapy, particularly RNA interference (RNAi), offers a promising approach to combating antibiotic resistance by targeting bacterial genes required for survival or resistance processes [130]. RNAi works by silencing certain mRNA molecules, which effectively prevents the creation of proteins that contribute to bacterial resistance. RNAi can prevent bacteria from expressing resistance features like efflux pumps or changed target sites, also decreasing the risk of off-target effects. Regarding bacterial infections, the use of short interfering silencing RNAs (siRNAs) showed effectiveness by inactivating bacterial invasion genes (such as SEC22A, Rab1B, and VPS33B) in *Yersinia ruckeri* [131], or by interfering with inflammatory and colonization-related genes in *H. pylori* (such as ureB and cagA) [132]. Concerning phenomena typically associated with antibiotic resistance, an in vitro and in vivo study by Yanagihara and colleagues (2006) demonstrated the advantage of using siRNA technology (targeting coagulase) to combat resistant *S. aureus* (MRSA) [133].

RNAi-based medicines are still in their early phases, but its focused method provides a fresh alternative to standard antibiotics, to which bacteria are increasingly resistant.

Another novel strategy could involve nanotechnologies, exploiting nanoparticles to deliver drugs directly to noxious bacteria. Nanoparticles, indeed, could represent a useful tool to improve targeted antibiotic delivery, increasing the medication’s potency and lowering the dosage needed [134]. The dimensions of nano-antibiotics may be below 100 nm, allowing them to penetrate bacterial cells more effectively compared to traditional formulations [134].

Basic tools for creating optimal pharmacological strategies to address the growing issue of antibiotic resistance have also been made available by bioinformatics, which covers the investigation and comprehension of macromolecules and their interactions. Machine learning could be helpful in the prediction of AMR and ABR, using whole-genome sequencing data instead of time-consuming experimental protocols [135]. Recently, Wu and colleagues (2023) proposed an Artificial Intelligence-powered strategy for the identification of resistance genes (ARG), based on the processing of protein sequences and the generation of vectors to represent each protein. These vectors can be used to develop models to identify antibiotic resistance and generate resistance categories [136].

## 5. Conclusions

Antibiotic resistance is a global emergency that can no longer be underestimated or ignored, and it is a problem that requires the immediate intervention of all nations. It is necessary to increase efforts in the field of research to ensure a future in which antibiotics will still be useful to combat infectious diseases. The risk is indeed that of going into a post-antibiotic era in which drugs will no longer be effective even for the most trivial infections. It is therefore important to involve all sectors at a global level, together with increasing the awareness of the population of the importance of antibiotics and their liability due to the presence of resistant pathogens. New approaches such as the use of natural polyphenols or biotechnological applications are encouraged to be developed in the fight against antibiotic-resistant bacteria.

## Figures and Tables

**Table 1:** Antibiotics’ primary targets and the corresponding key classes.

Target	Key Classes
Cell wall synthesis	β-lactams (penicillins, cephalosporins, monobactams, and carbapenems): they prevent the action of transpeptidase, the enzyme responsible for the formation of the bonds necessary for the structure of peptidoglycan [12,13,14]. Glycopeptides (vancomycin): they inhibit cell wall synthesis in bacteria by binding to the D-alanyl-D-alanine terminus of cell wall precursors [15].
Cytoplasmic membrane structure	Polymyxins (polymyxins B and E, also known as colistin): they can damage and break the membranes of the bacterium with their apolar tail [16].
Synthesis of nucleic acids	Quinolones (ciprofloxacin, nalidixic acid): they inhibit the action of two enzymes belonging to the class of topoisomerases (the DNA gyrase enzyme and topoisomerase IV), thus inhibiting DNA synthesis [17]. Rifamycins (rifampicin): they bind to the essential enzyme required for copying RNA from DNA, bacterial DNA-dependent RNA polymerase, preventing the start of RNA transcription, thereby stopping the synthesis of proteins required for bacterial development and propagation [18]. Nitroimidazoles (metronidazole): they disrupt DNA synthesis in anaerobic bacteria and certain protozoa, leading to bacterial death [19].
Folic acid metabolism	Sulfonamides (sulfamethoxazole): they inhibit folate synthesis, which is essential for bacterial growth and replication [20].
Protein synthesis	Aminoglycosides (gentamicin and tobramycin): they exert their function by binding to the 30S subunit, blocking the formation of the ribosome–mRNA complex [21]. Tetracyclines (doxycycline and minocycline): they can diffuse passively into the bacterial cell through pore channels and bind to the 30S subunit of the bacterial ribosome, inhibiting protein synthesis [22]. Oxazolidinones (linezolid): they inhibit protein synthesis by binding to the ribosomal 50S subunit [23]. Macrolides (erythromycin, azithromycin, and clarithromycin): these antibiotics inhibit protein synthesis by binding to the 50S subunit [24].

**Table 2:** Mechanisms of resistance to antibiotics developed by bacteria.

Mechanism	Main Examples
Limitation of drug absorption	It is a common mechanism in Gram-negative bacteria, which have an outer membrane (consisting of lipopolysaccharides (LPSs)) that protects the cytoplasmic membrane and prevents the entry of large polar molecules into the cell, while small polar molecules, such as antibiotics, penetrate through porins, which are transmembrane proteins. When porin channels undergo modifications or are not expressed at all, they can slow down or even block the entry of the antibiotic into the cell. If the outer membrane is changed or damaged, the antibiotic may have difficulty penetrating the cell, making it less effective [6].
Drug efflux	Efflux pumps are used by bacteria to transport toxic molecules out of the cell without modifying or degrading them. This mechanism using pumps has been detected in both Gram-negative and Gram-positive bacteria. Many antibiotics are actively transported out of the cell by bacterial efflux pumps, which could be specific to a particular antibiotic or different classes of antibiotics [34].
Changes in drug target	The alteration of the target leads to the loss of or decrease in the affinity of the drug for its target. It is sufficient to replace an amino acid with another to cause resistance, so-called point mutations. The target site can also be protected by removing the antibiotic from the binding site or by producing specific proteins that can compete for the same binding site as the antibiotic molecule, allowing for the survival of the bacterium. The function of the target site can also be performed vicariously by other sites of the protein, or another protein can even perform functions similar to those of the antibiotic target. These new structures have the benefit of not being sensitive to the administered antibiotic and, consequently, the bacterium survives and thrives [35].
Drug inactivation	Bacteria inactivate antibiotics by either chemically modifying them or destroying them. Bacteria generate enzymes that can bind to various chemical groups in the drug itself. This prevents the antibiotic from binding to the target in the bacterial cell. In general, acetylations and phosphorylations occur. The destruction of the drug concerns, for example, penicillins. Penicillin-resistant bacteria produce a particular enzyme, β-lactamase, which hydrolyzes the β-lactam ring of penicillins, inactivating them. The production of enzymes that inactivate antibiotics is one of the most common mechanisms of resistance [36].

**Table 3:** Summary of the main compound classes discussed in the text.

Class	Compounds	Main Source	Activity
Phenols	Pyrogallol, Pyrocatechol	Various plants, especially found in medicinal herbs	Effective against periodontitis-causing bacteria, Gram-positive, and Gram-negative bacteria [55].
Polyphenols	Resveratrol	Grapevines, pines, bananas, beans, and even pomegranates, peanuts, and soybeans	Inhibits biofilm formation, quorum sensing, and toxin synthesis; affects ATP synthase and cell division genes. Effective against *V. cholerae*, *E. coli*, and *S. aureus* [62,63,64,66,67].
	Curcuminoids	Turmeric (*Curcuma longa*)	Inhibit bacterial adhesion and growth; disrupt FtsZ protofilament assembly essential for cytokinesis. Effective against *S. aureus*, *E. coli*, *Enterococcus faecalis*, and *P. aeruginosa* [69,70].
Flavonoids	Anthocyanins, Catechins (e.g., Epigallocatechin Gallate)	Berries (anthocyanins), green tea (catechins)	Bacterial growth inhibition and biofilm activity interference; disruption of Na-K ATPase and protein synthesis [58]. Effective against *S. aureus* and *H. pylori* (resistant strains) [59].
Terpenoids	Carvacrol, Thymol, Geraniol, Farnesol, Salvipisone, Aethiopinone, Oleanolic Acid, Betulinic Acid, and Amyrin	Oregano, thyme, geraniums (*Salvia sclarea* for salvipisone), olive leaves (for oleic acid), birch trees (for betulinic acid), and Brazilian copal tree (for amyrin)	Inhibition of respiration; efflux pump disruption; show bactericidal/bacteriostatic effects. Effective against *MRSA*, *S. epidermidis*, Gram-positive, and Gram-negative bacteria [75,76,77,79,80,83].
Essential Oils	Basil, Thyme, Chamomile, Tea Tree	Basil, thyme, chamomile, tea tree	Antimicrobial effects against Gram-positive bacteria; highest activity from oregano and thyme essential oils [87].
Alkaloids	Berberine, Sanguinarine, Piperine, Capsaicin	Berberis species (berberine), *Macleaya cordata* (sanguinarine), black pepper (piperine), chili peppers (capsaicin)	Inhibit cell division, respiration, and enzymes; disrupt membranes and biofilms. Effective against *MRSA*, *S. aureus*, and *Salmonella* spp. [90,91,92,93,94,95].
Phenolic Acids	Caffeic Acid, Gallic Acid, Ferulic Acid	Coffee beans (caffeic acid), oak galls (gallic acid), rice, wheat, oats, peanuts (ferulic acid)	Disrupt cell wall; enhance antibiotic effects (e.g., quinolones); induce ROS. Effective against *S. aureus*, *E. coli*, and *A. baumannii* [71,72,73,74].
Secoiridoids	Oleocanthal, Oleacein	Extra virgin olive oil	Inhibit biofilms and reduce bacterial growth and adhesion. Effective against *P. aeruginosa*, *Chlamydia trachomatis*, *E. coli*, *S. aureus*, and *K. pneumoniae* [97,99].
Polyamines		Various plant and animal sources	Increase sensitivity of *P. aeruginosa* to various antibiotics [100].
Isothiocyanates	Sulforaphane	Broccoli, cabbage, kale	Antibacterial against *H. pylori* in vitro and in vivo [101].
Glucosides	Glycyrrhizin	Licorice root (Glycyrrhiza glabra)	Effective against *E. coli* [103].
Thiosulfinates	Allicin	*Garlic* (Allium sativum)	Broad spectrum; disrupts bacterial enzymes by binding thiol groups. Effective against Gram-positive and Gram-negative bacteria, including resistant strains [104].
